# Analysis of Pigeon (*Columba*) Ovary Transcriptomes to Identify Genes Involved in Blue Light Regulation

**DOI:** 10.1371/journal.pone.0143568

**Published:** 2015-11-24

**Authors:** Ying Wang, Jia-tong Ding, Hai-ming Yang, Zheng-jie Yan, Wei Cao, Yang-bai Li

**Affiliations:** College of Animal Science and Technology, Yangzhou University, Yangzhou, Jiangsu Province, 225009, P. R. China; Oregon State University, UNITED STATES

## Abstract

Monochromatic light is widely applied to promote poultry reproductive performance, yet little is currently known regarding the mechanism by which light wavelengths affect pigeon reproduction. Recently, high-throughput sequencing technologies have been used to provide genomic information for solving this problem. In this study, we employed Illumina Hiseq 2000 to identify differentially expressed genes in ovary tissue from pigeons under blue and white light conditions and *de novo* transcriptome assembly to construct a comprehensive sequence database containing information on the mechanisms of follicle development. A total of 157,774 unigenes (mean length: 790 bp) were obtained by the Trinity program, and 35.83% of these unigenes were matched to genes in a non-redundant protein database. Gene description, gene ontology, and the clustering of orthologous group terms were performed to annotate the transcriptome assembly. Differentially expressed genes between blue and white light conditions included those related to oocyte maturation, hormone biosynthesis, and circadian rhythm. Furthermore, 17,574 SSRs and 533,887 potential SNPs were identified in this transcriptome assembly. This work is the first transcriptome analysis of the *Columba* ovary using Illumina technology, and the resulting transcriptome and differentially expressed gene data can facilitate further investigations into the molecular mechanism of the effect of blue light on follicle development and reproduction in pigeons and other bird species.

## Introduction

The White King pigeon (*Columba*) is an important commercial meat pigeon that has become popular in China in recent years [1–2]. Pigeons were probably domesticated in the Mediterranean region at least 3,000–5,000 years ago and possibly even earlier as a food source [3]. Paired pigeons lay only two eggs in a laying period, and newly hatch squabs are fed with crop milk from their parents, which is unique among birds [4]. Furthermore, the duration from egg laying to foraging ability of squabs is at least 38 days. Thus, the low productivity of the pigeon breeding industry hinders its development and needs to be improved.

Light is a major environmental factor affecting the reproductive activity of birds. Different light wavelengths have varying stimulatory effects on the retina and pineal cells of birds, resulting in behavioral changes that affect their physiology and reproduction [5]. Previous studies of the influence of light wavelength on physiological mechanism and its reproductive performance have been conducted in birds. McGinnis (1967) reported the chicken pullets were stimulated early reproductive activity by blue light, while Woodard et al. (1969) confirmed fertility rate of eggs under blue light was significantly lower than under white light [6–7]. Yadav et al. (2015) suggested that long-term exposure to blue light at low intensity may induce gonadal regression, even under long-day conditions [8]. A previous study from our group affirmed that monochromatic light influences the reproductive performance of pigeons [9]. In addition to the effect of light on reproductive activity, Halevy (2006) and Liu et al. (2010) found a stimulatory effect of green light on skeletal muscle development in chicks in ovo [10–11], and Xie et al. (2008) and Sadrzadeh et al. (2011) reported that monochromatic light affected immune function in broiler chickens [12–13]. However, the molecular mechanism by which monochromatic light influences physiological function in pigeons is poorly understood and needs further exploration.

Transcriptome sequencing can be used to discover genes that are functionally active and participate in specific biological processes [14–15]. In particular, Illumina sequencing technology can be used for gene identification with confirmed reliability [16]. Despite improvement in sequencing tools, sequence databases that provide a molecular understanding of bird physiology are still lacking. In the present study, we employed Illumina sequencing technology and *de novo* assembly to produce a transcriptome of *Columba* ovary tissue under blue and white light conditions and to annotate the expressed genes without related genome information. Our results not only provide reference information for the molecular mechanism governing reproductive performance under blue light, but also investigate the simple sequence repeats (SSRs) and single nucleotide polymorphisms (SNPs), which are valuable for further research on pigeons and related species.

## Experimental Section

### Ethics approval

This study was reviewed and approved by the Institutional Animal Care and Use Committee (IACUC) of the Department of Animal Science and Technology, Yangzhou University and was performed in accordance with the Regulations for the Administration of Affairs Concerning Experimental Animals (China, 1988). All pigeon procedures were performed according to the Standards for the Administration of Experimental Practices (Jiangsu, China, 2008).

### Pigeon rearing and sample preparation

White King pigeons were raised in an isolated loft at the College of Animal Science and Technology, Yangzhou University, Yangzhou. Seventy-two birds were divided into white and blue light groups, with three subgroups, the experiment was 6 months in length. Pigeons were provided with food and water *ad libitum*. Birds were exposed to white light (400–760 nm) or blue light (480 nm), received 15 h of light exposure (15 h light, 9 h dark). The intensity of light was 15.20 ± 0.65 lux as measured using a TES-1336A light meter (TES Electrical Electronic Crop., Taipei, China).

Six female birds with similar body weights (mean weight: 557.02 g) and similar physiological periods (i.e., the day after the second egg was laid) were anesthetized with sodium pentobarbital at a dosage of 2.5 mg/100 g body weight. All efforts were made to minimize distress. Ovarian samples were rapidly collected, flash frozen in liquid nitrogen, and stored at -80°C.

### Total RNA extraction and cDNA library preparation

Ovary tissues of three individuals from two groups were subjected to RNA extraction. Total RNA was extracted from the collected ovaries using TRIzol reagent (Invitrogen, Carlsbad, CA, USA) following the manufacturer’s protocol. The samples for transcriptome analysis were prepared using Illumina’s kit following the manufacturer’s instructions. The quantity, purity, and integrity of RNA were measured using a 2100 Bioanalyzer (Agilent Technologies, Wilmington, DE, USA). Oligo (dT) magnetic beads were used to purify 6 μg of total RNA, furthermore, ovary tissues from 6 pigeons were used to establish libraries respectively. Short fragments (approximately 200 bp) were obtained and used as templates for first-stand cDNA synthesis using random hexamer primers. The second strand was synthesized using buffer, dNTPs, RNase H, and DNA polymerase I. The double-stranded cDNA was purified using the Qiaquick PCR extraction kit (Qiagen, Hilden, Germany) and eluted with elution buffer for end-repair and poly(A) addition. Finally, sequencing adaptors were ligated onto the fragments. The cDNA library was sequenced on the Illumina sequencing platform, and raw reads were generated using the Solexa pipeline according to the manufacturer’s recommendations.

### *De novo* transcriptome assembly

Adaptor sequences and low-quality sequences (threshold quality, 20; window size, 5 bp; threshold length, 35 bp) were filtered. The paired-end sequencing strategy was used to better assemble the transcriptome *de novo*. *De novo* transcriptome assembly was performed using the Trinity program (version r2013/11/10) [17], and the Trinities were clustered into unigenes using TGICL tools [18].

### Functional annotation of unigenes

The assembled unigenes were searched against the BLAST NR protein sequence database (http://blast.ncbi.nlm.nih.gov), the Swiss-Prot database (http://www.expasy.ch/sprot), and the KEGG database (http://www.genome.jp/kegg) using the BLASTx algorithm. A typical cut-off value of E<1e^-5^ was used. The unigenes were sorted to recover proteins with the most similarity to the unigenes with putative functional annotations. GO annotation of the unigenes was obtained at the second level according to biological process, component function, and cellular component. Furthermore, eukaryotic orthologous groups were used to predict and classify unigene functions.

### Molecular markers detection

MIcroSAtellite (http://pgrc.ipk-gatersleben.de/misa) was used to identify putative SSRs in the unigenes from the assembled transcripts. The minimum number of repeat units for mono-, di-, tri-, tetra-, penta- and hexa-nucleotide motifs were set as 10, 6, 5, 5, 5 and 5, respectively. Samtools and bcftools were applied to identify putative SNPs in this transcriptome.

### Validation of RNA-seq results by qRT-PCR

Total RNA was isolated by TRIzol reagent (Invitrogen), and the concentration of RNA was diluted to 1 μg/μl to be used for first-strand cDNA synthesis using the Fast Quant RT Kit (Tiangen). qRT-PCR was performed using SuperReal PreMix (SYBR Green, Tiangen) according to the manufacturer’s protocol. To validate the assembled unigenes, 8 unigenes were selected for qRT-PCR (S1 Table). All measurements were conducted in triplicates. The pigeon glyceraldehyde-3-phosphate dehydrogenase (GAPDH) gene was used as an internal control. The 2^-ΔΔCT^ method was used to analyze relative RNA expression [19].

## Results and Discussion

### De novo transcriptome sequencing and assembly

**Fig 1:**
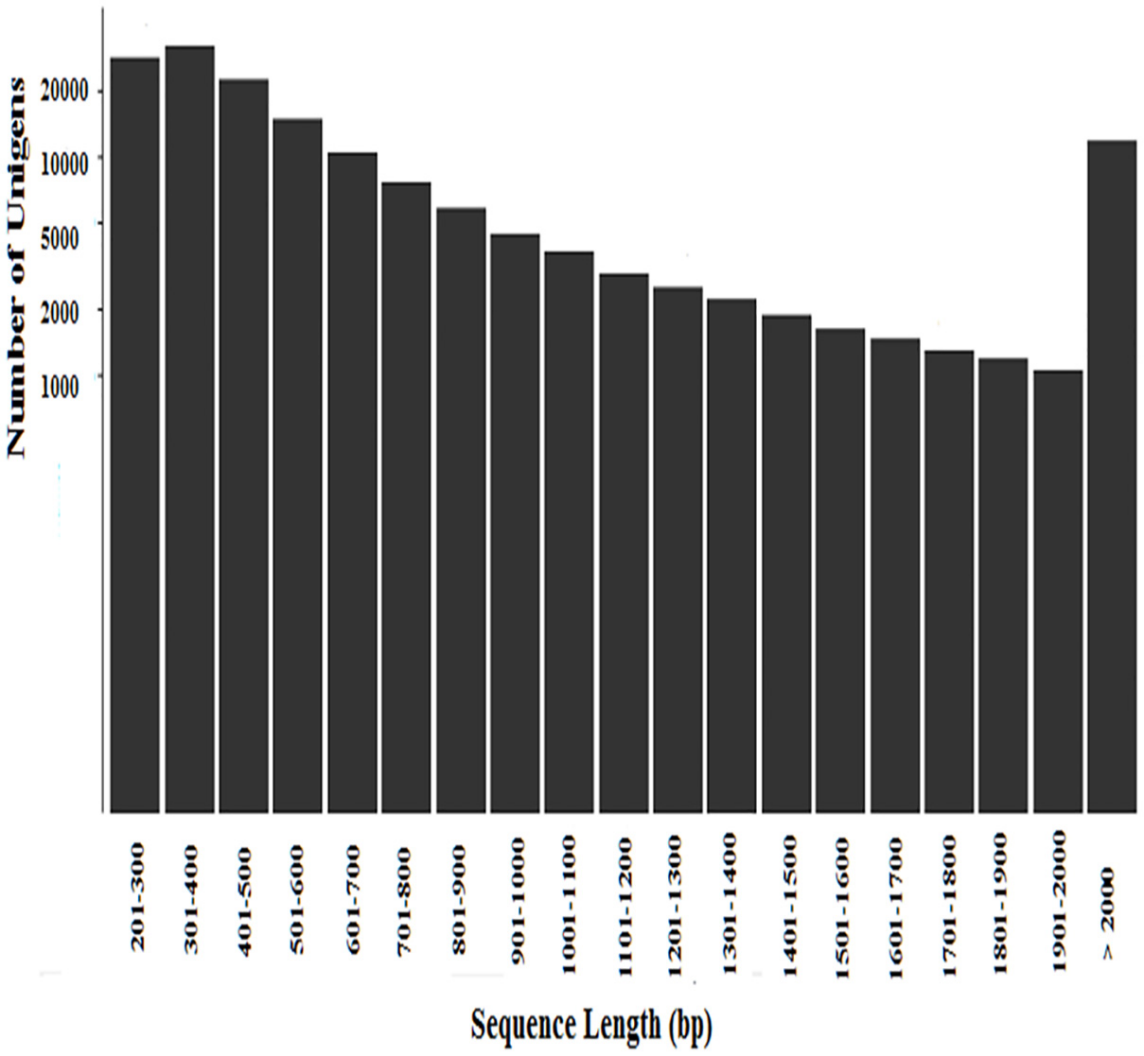
Length distribution and frequency of unigenes in *Columba*.

From our reproductive data, the total egg production of pigeons (32.67±1.67 monthly) under blue light which was significantly lower than white light (68.16±3.45 monthly). This results induced us to explore differences in pigeon ovary transcriptome under blue and white light conditions, three pigeons under blue light (B1, B2, and B3) and three pigeons under white light (W1, W2, and W3) were selected for analysis. The cDNA of pigeons was sequenced by Illumina Hiseq 2000, and transcriptome sequencing generated 14.07 million and 11.90 million reads from ovaries under blue and white light conditions, respectively. Detailed results of sequencing and assembly are shown in Table 1. Trinity software was applied to assemble the reads into a transcriptome, as no reference genome is yet available for *Columba* [20–21]. The numbers of clean reads in the ovary transcriptome libraries for blue and white light conditions were 17.39 Gb and 14.69 Gb, respectively. We obtained 157,774 unigenes (mean length: 790 bp) with an N50 of 1,108 bp. The length of the unigenes ranged from 201 to 17,581 bp, and the lengths of 11,781 unigenes greater than 2,000 bp (Fig 1). Notably, the unigenes obtained in the present study were longer than those obtained in previous studies.

**Table 1:** Summary of Illumina sequencing and transcriptome assembly.

Condition	Sample name	Raw reads	Clean reads	Clean bases (bp)	Valid rate (%)	Q30 (%)	GC content (%)
Blue light	B1	41550778	41229474	5140724575	98.97	94.23	53.00
Blue light	B2	55086542	54601944	6810865040	98.88	93.86	53.00
Blue light	B3	43980608	43620184	5437497814	98.90	94.00	53.00
White light	W1	42935054	42523434	5298145890	98.71	93.79	55.00
White light	W2	33365916	33090892	4132171509	99.07	93.2	54.00
White light	W3	42670976	42283004	5258718979	98.59	92.07	55.00

Our analysis indicated that, in all samples, 84.41% of the left and 91.02% of the right reads could be mapped back to the assembled transcriptome, with 65.53% of proper pairs mapped for a representative sample. Unmapped sequences were due to read orphans, poor-quality reads, or incomplete transcripts. Until now, no standard criteria have been applied to evaluate the quality of transcriptome assemblies [22]. Our results indicate that the Illumina-generated dataset has high reliability and covers most transcriptome sequences, which can be valuable for further research. The sequences of unigenes were deposited in the NCBI Transcriptome Shortgun Assembly Sequence Database (B1: SRR2094734, B2: SRR2094746, B3: SRR2094764; W1: SRR2094777, W2: SRR2094789, W3: SRR2094799.).

### Functional annotation and classification

**Fig 2:**
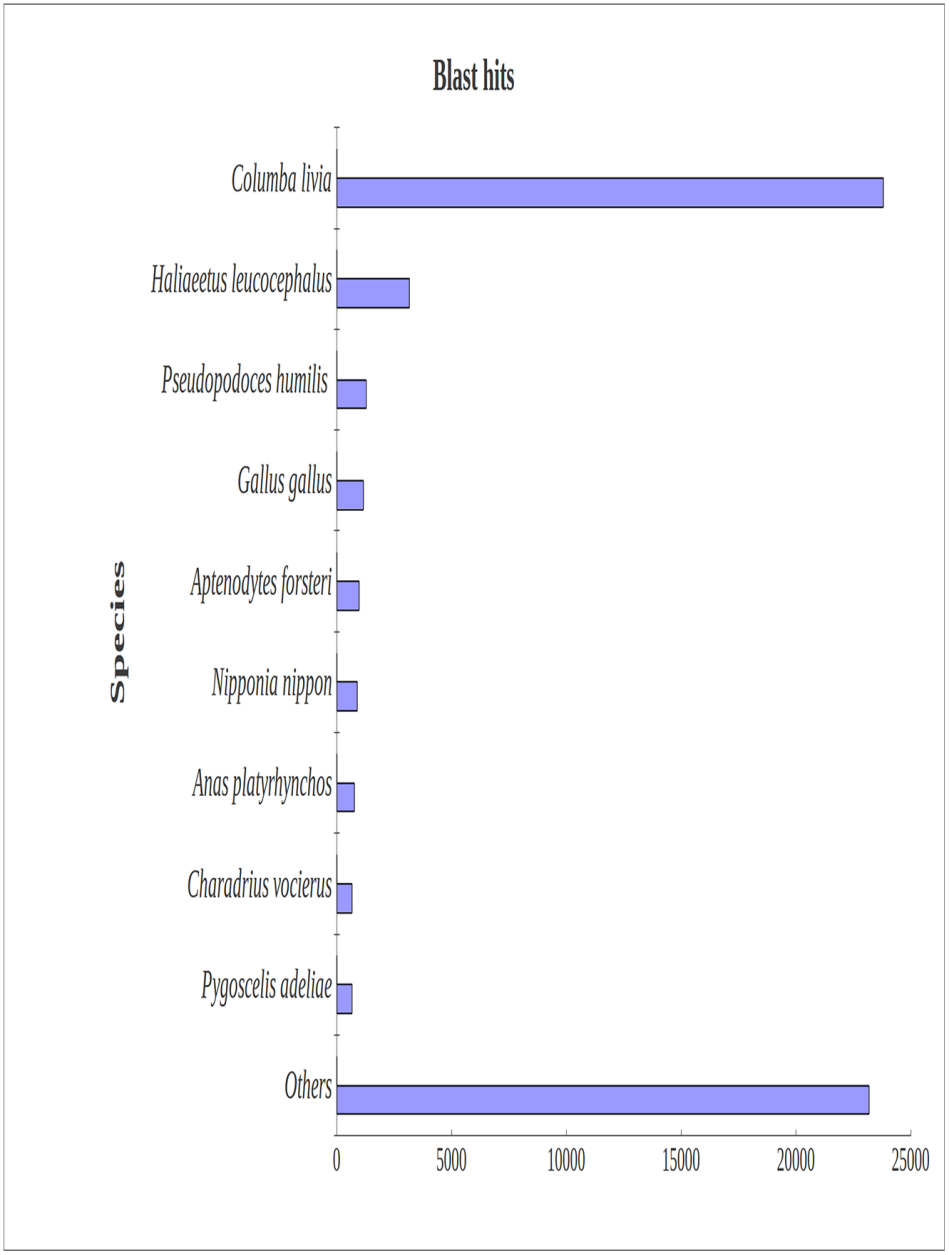
Species distribution of BLASTx matches for ovary transcriptome unigenes. More than 40% of identified transcripts had the highest homology with *Columba livia*, and about 6% of top hits matched *Haliaeetus leucocephalus*.

Blast2GO was used to annotate the unigenes [23]. We carried out a BLASTx search against NR (non-redundant) and Swiss-prot protein databases with a cut-off E-value of 10^−5^ or lower. Short assembled Trinity transcripts were difficult to match with known genes; 56,530 unigenes (35.83% of all distinct sequences) and 45,797 unigenes (29.03% of all distinct sequences) were determined as significant hits in the NR and Swiss-prot databases, respectively (Table 2). Many sequences did not have BLASTx hits. Most (42.10%) BLASTx-hit transcripts matched *Columba livia*, followed by *Haliaeetus leucocephalus*, *Pseudopodoces humilis*, *Gallus gallus*, and *Aptenodytes forsteri* (Fig 2).

**Table 2:** List of annotations.

Annotation database	Annotation number	Percent of annotation (%)
Total unigenes	157774	100
NR	56530	35.8297
Swiss-prot	45797	29.0270
COG	34655	21.9650
KEGG	18203	11.5374
GO	18833	11.9367

NR, non-redundant; COG, cluster of orthologous group; KEGG, Kyoto Encyclopedia of Genes and Genomes; GO, gene ontology.

**Fig 3:**
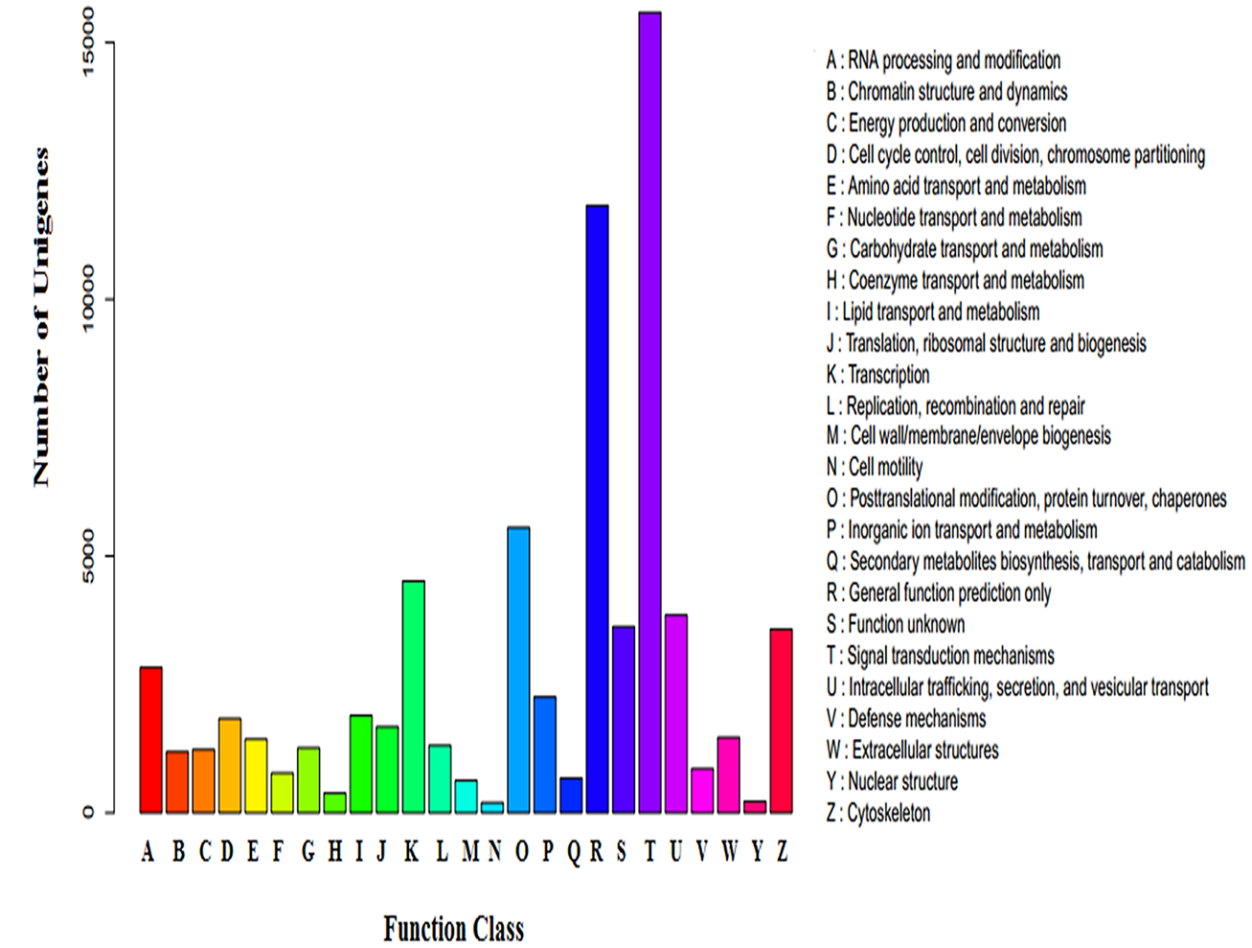
COG classifications. A total of 34,655 sequences were grouped into 25 COG categories.

The cluster of orthologous group (COG) classifications of annotated sequences were used to determine the effectiveness of the annotation process and the completion of the transcriptome library. A total of 34,655 sequences were aligned to the COG database (Fig 3). The cluster for ‘signal transduction mechanisms’ represented the largest group (15,587, 44.98%), followed by ‘general function prediction’ (11,827, 34.13%) and ‘posttranslational modification, protein turnover, chaperones’ (5555, 16.03%). ‘Nuclear structure’ (220, 0.0063%) and ‘cell motility’ (198, 0.0057%) were the smallest groups.

**Fig 4:**
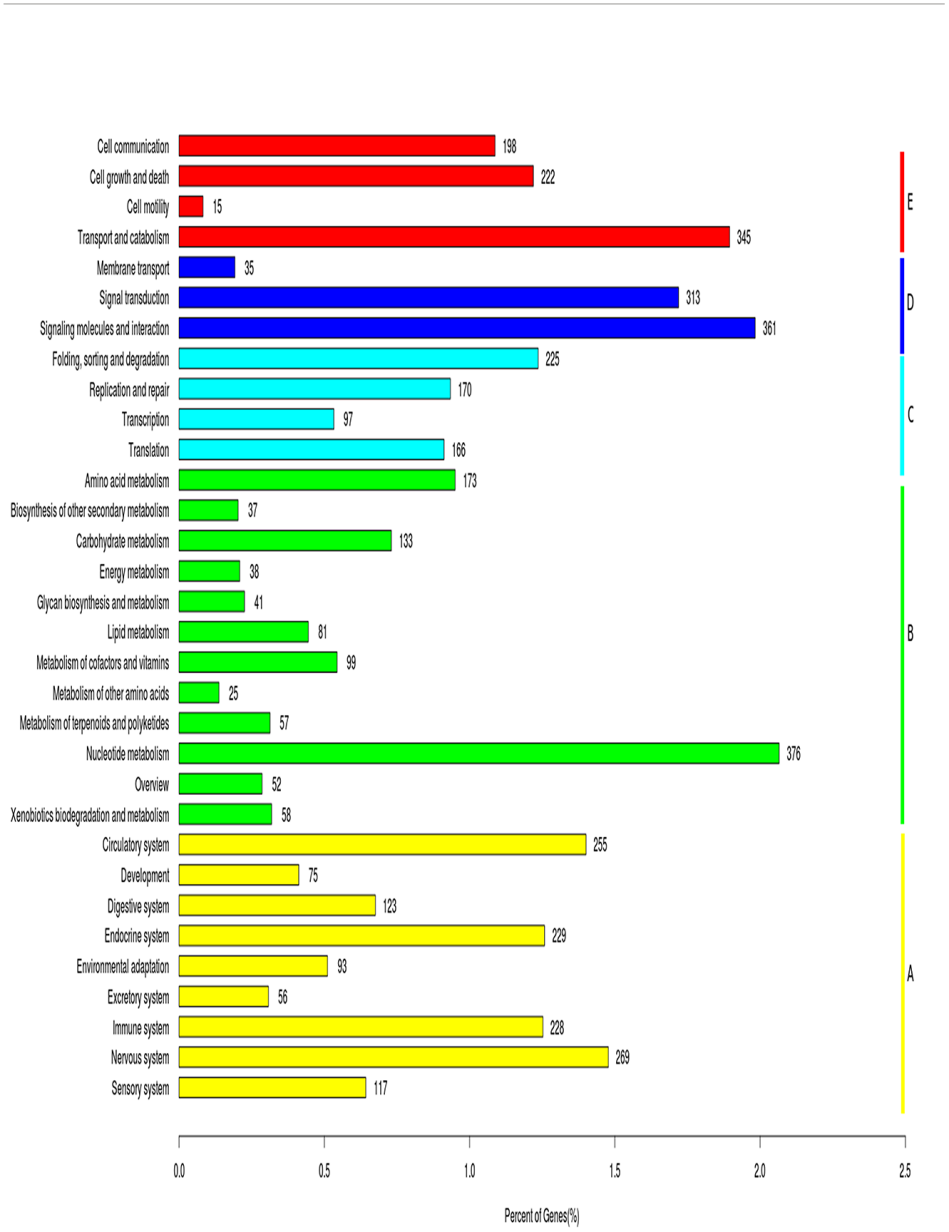
Pathway assignment based on the KEGG database. Classification based on (A) organismal system categories, (B) metabolism categories, (C) genetic information processing categories, (D) environmental information processing categories, and (E) cellular process categories.

All unigenes were mapped to reference canonical pathways in the Kyoto Encyclopedia of Genes and Genomes (KEGG) database (Fig 4) [24]. A total of 18,203 unigenes were matched to 356 KEGG pathways (S2 Table). The most highly enriched pathways were those related to PI3K-Akt signaling (n = 998), MAPK signaling (n = 801), and focal adhesion (n = 772). Enriched pathways also included those involved in reproduction and circadian rhythm, such as ovarian steroidogenesis, Wnt signaling, estrogen signaling, and circadian rhythm and entrainment [25–26].

**Fig 5:**
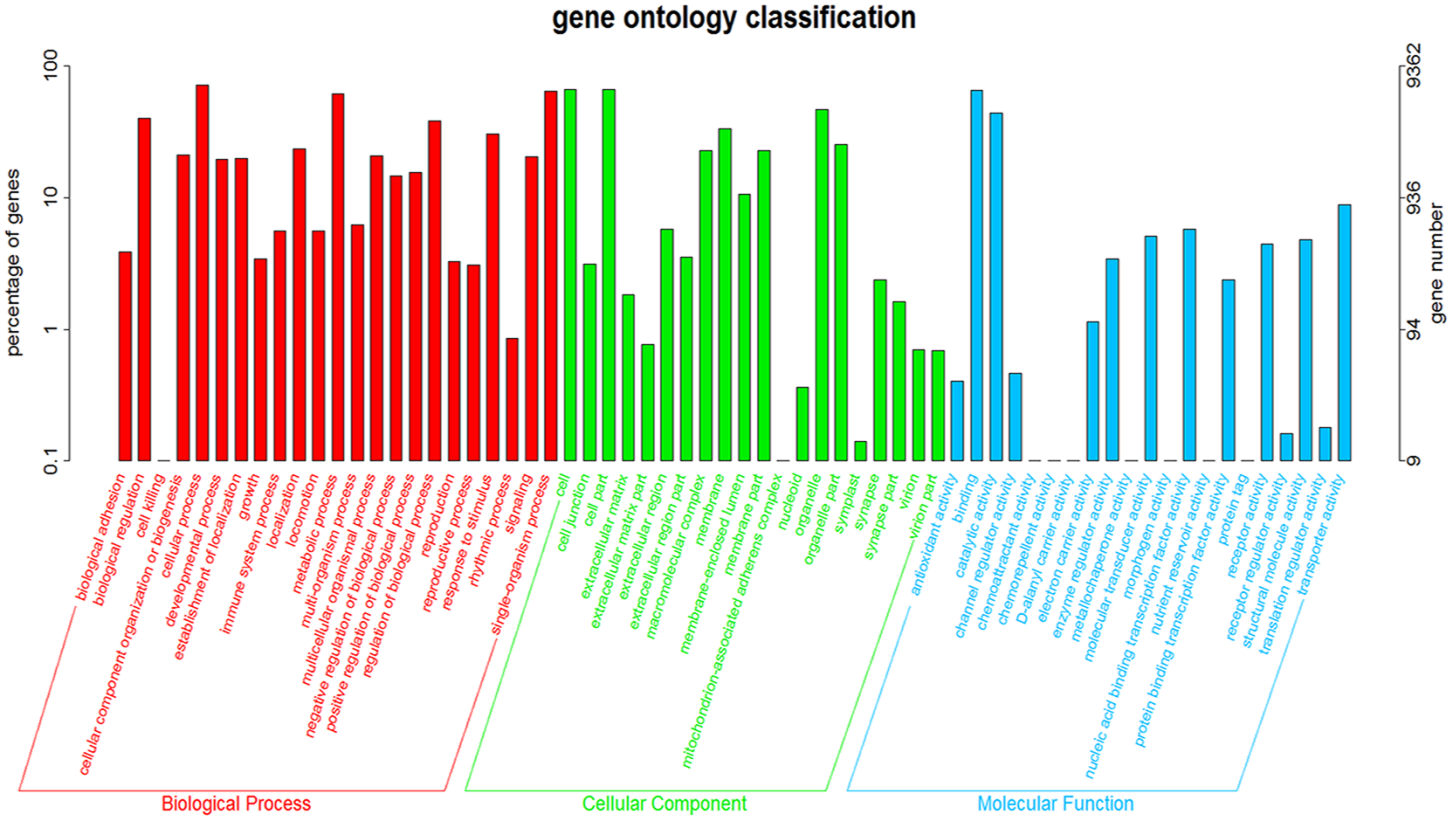
GO classification map. The x-axis indicates the next level GO term of the three GO categories: biological process, cellular component, and molecular function.

When gene ontology (GO) assignment programs were applied for functional categorization, a total of 18,833 unigenes were classified into 63 functional groups (Fig 5) [27]. Biological process, cellular component, and molecular function were the three main ontologies. As expected, the reproductive process, reproduction, and the rhythmic process were found among the biological process categories (S3 Table). This GO assignment results are similar to the previously sequenced *Anser cygnoides* ovary transcriptome [28]. The KEGG and GO annotations are valuable for identifying potential genes from the vast transcriptome database, providing a helpful information on the mechanisms by which light wavelength may influence the bird ovary.

### Analysis of differentially expressed genes (DEGs)

The light spectrum affects the reproduction of pigeons due to changes in reproductive processes and circadian rhythm [10, 29]. Although the effect of monochromatic light on the reproductive performance and growth of birds have been widely studied, the molecular mechanisms regulating biosynthesis and reproductive function remain unknown. We used the reads per kilobase per million method to calculate gene expression filtered by a false discovery rate of <1 and log2 (fold change) of >1 [30]. A total of 6,831 DEGs were identified between blue and white light conditions, with 3,305 up-regulated and 3,526 down-regulated genes. These genes include those involved in hormone synthesis, oocyte meiosis, and circadian rhythm (S4 Table). As the number of unigenes with no homologs in the NR database was 45%, this suggests that some of the DEGs may be expressed specifically in the pigeon ovary and involved in ovulation. In our study, a number of genes involving in the circadian rhythm, and genes regulating the synthesis and metabolism of hormones and vital components for oocyte maturation. The further studies are necessary to identify these unknown genes which will facilitate molecular mechanism of blue light transduction in pigeons.

The circadian clock, regulates the circadian rhythm [31], and circadian rhythm is a 24 hr rhythms of behavior and other physiological function which are based upon an endogenous self-sustained oscillation [32]. Wunderer et al. (2013) suggested that clock genes and their protein products may be directly involved in the photoperiod-dependent regulation and adaptation of hormone synthesis and release [33]. The pathway of circadian entertainment controls the pathway of circadian rhythm, light induces the presynaptic retinal ganglion cells (RGC) neurons and postsynaptic suprachiasmatic nucleus (SCN) neurons, which trigger the clock genes immediate early genes and the initial of circadian rhythm. The PER and CRY synthesize heterodimer which works as negative component while the BMAL1 and CLOCK heterodimer is positive component [34–35], PER/CRY heterodimers inhibit CLOCK and BMAL1 expression, forming a loop or cycle of CLOCK/BMAL1-PER/CRY [36]. The Dec, Ror and CK1 genes regulate the circadian rhythm which were found in this transcriptome. AMPK modulated the degradation of CRY, was also identified in this transcriptome [37]. Furthermore, our previous studies showed the BMAL1 which was a core component of the circadian rhythm, correlated with birth rate of pigeons under the monochromatic lights supplement [9].

Steroid hormones, such as estrogen, androgen and progestin, have been studied in pigeons, regulated vitellogenesis, incubation, and oviduct development [38–41]. The enzymes involved in steroid biosynthesis pathway are being recognized as important target for endocrine-disrupting, which would impair reproduction [42], the 3β-hydroxysteroid dehydrogenase (3-β HSD) and aromatase cytochrome P450 enzymes which are essential for the biosynthesis of all classes of steroid hormones, were detected in this transcriptome. All active steroid hormones need to be converted from 3-β HSD precursors to hormonally active 3-ketosteroid, which is Δ^5^–3β-hydroxysteroids converted into Δ^4^-3-ketosteroids [43], a reaction step catalyzed by 3-β HSD [44]. P450_arom_ catalyzes the conversion of androgens to estrogen, which is a key step in estrogen biosynthesis [45–46]. Furthermore, placental P450_arom_ converts androstenedione (Δ^4^A) and testosterone (T) derived from fetal and maternal adrenal dehydroepiandrosterone sulfate (DHEA-S) to estrone (E_1_) and estradiol (E_2_) [47]. Apart from the genes involved in steroid hormone biosynthesis, genes encoding their receptor were also detected, such as estrogen receptor β (ERβ), mice lacking ERβ have fewer and smaller litters than wild-type mice [48]. The expression of ERβ and 3-β HSD in ovary was significantly lower in blue light, which in accord with the egg production of this experiment.

Oocyte maturation, immature oocytes become fertilizable eggs through meiotic maturation [49], in Xenopus oocytes, maturation is thought to be initiated by steroid hormone progesterone [50], the progesterone-mediated oocyte maturation pathway was found here. There is some evidence that progesterone receptor in oocytes is a membrane-bound receptor, possibly coupled to heterotrimeric G-proteins, which inhibit adenylate cyclase [51]. Gβγ-subunit activates meiotic maturation, which may be mediated by PI3K activation. Previous study have showed phosphatidylinositol 3-kinase (PI3K) is known to play critical roles in signal transduction processes related to a variety of cellular activities [52], which participate in mouse meiotic maturation and also implicate in progesterone-induced maturation [53]. Furthermore, class 1A PI3K catalytic subunits (locus name PIK3c) are associated with a regulatory subunit (PIK3r) which participate in phosphorylation reaction [54], both genes were identified in this transcriptome. The gene protein kinase Mos and anaphase-promoting complex regulated the oocyte maturation were identified through the Illumina analysis [55–56], which will enable us to inspect the molecular mechanism of oocyte maturation which affect the reproductive process of pigeon.

In this *de novo* transcriptome assembly, we obtained a number of genes involved in the regulation of light spectra (blue and white light) in pigeons, most of these genes were discovered in pigeons for the first time, such as RORβ, 3β-HSD and CDC27. Further studies are required to elaborate their roles in reproductive process under the blue light in pigeons.

### DEGs in the ovary under blue and white light conditions

As it is important to understand the mechanisms of the effect of light wavelengths on ovulation, we characterized annotated DEGs related to ovulation, which included genes involved in ovarian steroidogenesis (*bone morphogenetic protein 15* (*BMP15*), *3-β-hydroxysteroid dehydrogenase* (*3β-HSD*)), oocyte meiosis (*cell division cycle 27* (*CDC27*) and *mitotic spindle assembly checkpoint*), and cell cycle (*transforming growth factor β2* (*TGF-β2*), *E2F transcription factor 1* (*E2F1*)). In addition to genes related to ovulation, we also characterized several genes previously found to play a role in the effects of blue light, including *estrogen receptor β* (*ERβ*), *mitogen-activated protein kinase kinase kinase 1* (*MAP3K1*), *nuclear receptor RORβ*, and *melatonin receptor*.

*MAP3K1*, an important component of the MAPK pathway, cooperates with other factors in steroid-dependent transcription [57]. In the present study, *MAP3K1* expression in the ovary was up-regulated under blue light, indicating its potential involvement in the effects of monochromatic light. Previous studies show that *BMP15* enhances oocyte development and regulates oocyte developmental programming, and *BMP15* mutation causes monoovulatory cycles in humans and reduces ovulation rate in mice [58–59]. In the present study, *BMP15*, *ERβ*, and *3β-HSD* gene expression in the ovary were down-regulated under blue light, which is also consistent with the reproduction data in our experiment, which is in accordance with the results of Foss and White (1983), showing that a long wavelength of light might increase egg production in brown egg laying hens [60].

*CDC27* is a part of the anaphase-promoting complex, which plays a vital role in mitosis [61]. *E2F1* is thought to act as a transcriptional activator of progression through the G1/S transition, and loss of this factor abolishes the ability of mouse embryonic fibroblasts to enter S phase and progress through the cell cycle [62–63]. *TGF-β2* also affects cell cycle. In the present study, we observed changes in the expression of *TGF-β2* and *E2F1* under blue and white light conditions, which are in accordance with the change of these genes in the cell cycle pathways. Also, as *RORβ* contributes to the peripheral circadian clock [64–65], the higher expression of *RORβ* observed in the present study suggests that circadian clock genes involved in the blue light mechanism on pigeons.

To validate the expression profiles obtained from Illumina sequencing analysis, eight DEGs chosen for their closely relation to ovulation and circadian rhythm were analyzed by quantitative real-time polymerase chain reaction (qRT-PCR) analysis. We observed that the trend of DEGs expressions were similar to the sequencing data (Table 3), providing further evidence of the credibility of the sequencing database.

**Table 3:** Real-time PCR confirmation of DEGs in ovaries between blue and white light conditions (log_2_fold-change).

Sequence ID	Gene	Illumina sequencing	Real-time PCR
CL15329Contig1	estrogen receptor β	-1.50	-1.18
CL10084Contig1	cell division cycle 27	4.48	0.81
CL3334Contig2	mitogen-activated protein kinase kinase kinase 1	2.58	1.47
CL9880Contig1	bone morphogenetic protein 15	-1.82	-0.92
CL12107Contig1	transforming growth factor β2	2.15	1.77
CL2Contig675	nuclear receptor RORβ	-1.72	-0.67
CL8711Contig1	3-β-hydroxysteroid dehydrogenase	-2.33	-1.60
CL8514Contig1	E2F transcription factor 1	-1.76	-0.97

### Putative molecular markers

A total of 533,887 potential SNPs were identified among all of the unigens using the Poly Bayers (Table 4), including 400,607 (75.04%) transitions. The greatest amount of transitional base changes (53,809 A-G, 56,616 G-A, 72,756 C-T and 32,829 T-C) were from blue light, while the white light samples had (41,726 A-G, 51,989 G-A, 62,991 C-T and 27,891 T-C). 133,280 (24.96%) tranversions, including 3.43% A-C, 2.43% A-T, 2.94% C-A, 4.68% C-G, 3.41% G-C, 4.16% G-T, 1.68% T-A and 2.22% T-G. To determine the quality of the unigene database, 157,774 unigenes were assembled in this study to detect potential SSRs. A total of 17,574 potential SSRs were identified, which included two types of mononucleotide, four types of dinucleotide, ten types of trinucleotide and tetranucleotide, pentanucleotide, hexanucleotide SSRs. Mononucleotides SSRs were the most abundant microsatellite repeats unites (11,221, 63.85%), dinucleotide SSRs were second (2673 15.21%), followed by trinucleotide (3314, 18.86%), tetranucleotide (308, 1.75%), pentanucleotide (45, 0.26%), hexanucleotide (13, 0.07%) (Table 5). It was obvious that A/T accounted for 74.21% of the mononucleotide SSRs, AC/GT accounted for 44.56% of the dinucleotide SSRs, AGG/CCT accounted for 38.71% of the trinucleotide SSRs. The validation of the putative SNPs not only showed the utility of Illumina sequence for SNPs, but also identified a large number of SNPs and SSRs. This huge data will provide a valuable resources for the further genetic study of pigeons.

**Table 4:** Summary of single nucleotide polymorphisms (SNPs) of transcriptomic data from *Columba*.

SNP Type	Blue	White	Total
Transition	216010	184597	400607
A-G	53809	41726	95535
G-A	56616	51989	108605
C-T	72756	62991	135747
T-C	32829	27891	60720
Transversion	74836	58444	133280
A-C	10545	7778	18323
A-T	7472	5504	12976
C-A	8515	7176	15691
C-G	14036	10961	24997
G-C	10053	8162	18215
G-T	12634	9592	22226
T-A	5054	3932	8986
T-G	6527	5339	11866
Total	290846	243041	533887

**Table 5:** Summary of SSRs identified from the ovary transcriptomme of *Columba*.

SSR type	Repeats	Total number	Proportion of total SSRs(%)
Mononucleotide	Total	11221	63.85
	A(T)	8327	47.38
	C(G)	2894	16.47
Dinucleotide	Total	2673	15.21
	AC(GT)	1191	6.78
	AG(CT)	535	3.04
	AT(AT)	932	5.30
	CG(CG)	15	0.09
Trinucleotide	Total	3314	18.86
	AAC(GTT)	155	0.88
	AAG(CTT)	210	1.19
	AAT(ATT)	251	1.43
	ACC(GGT)	213	1.21
	ACG(CGT)	10	0.06
	ACT(AGT)	13	0.07
	AGC(CTG)	753	4.28
	AGG(CCT)	1283	7.30
	ATC(ATG)	177	1.01
	CCG(CGG)	249	1.42
Tetranucleotide	Total	308	1.75
Pentanucleotide	Total	45	0.26
Hexanucleotide	Total	13	0.07

## Conclusions

In this study, we generated the first reference sequence for *Columba* by RNA sequencing of ovarian tissue under blue or white light conditions and cloud-based *de novo* transcriptome assembly. We obtained 157,774 unigenes with 56,530 sequences with a cut-off E-value greater than 10^−5^. This transcriptome database provides genomic information on the mechanisms of ovulation under different light wavelengths in pigeons. Considering the reliability of Illumina sequencing analysis for the detection of DEGs, potential SSRs and SNPs, our findings should be useful for future functional studies of genes involved in bird reproduction.

## Supporting Information

S1 Table Primers used for the validation of DEGs by qRT-PCR. (XLS)

S2 Table Detailed information of KEGG pathway analysis. (XLS)

S3 Table Significantly enriched GO terms in DEGs. (XLS)

S4 Table Candidate genes involved in the regulation of blue light in Columna livia. (XLS)
